# The Effect of Human Immunodeficiency Virus on Hepatitis B Virus Serologic Status in Co-Infected Adults

**DOI:** 10.1371/journal.pone.0008687

**Published:** 2010-01-13

**Authors:** Michael L. Landrum, Ann M. Fieberg, Helen M. Chun, Nancy F. Crum-Cianflone, Vincent C. Marconi, Amy C. Weintrob, Anuradha Ganesan, Robert V. Barthel, Glenn Wortmann, Brian K. Agan

**Affiliations:** 1 Infectious Disease Clinical Research Program, Uniformed Services University of the Health Sciences, Bethesda, Maryland, United States of America; 2 Infectious Disease Service, San Antonio Military Medical Center, Fort Sam Houston, Texas, United States of America; 3 Division of Biostatistics, University of Minnesota, Minneapolis, Minnesota, United States of America; 4 Department of Defense HIV/AIDS Prevention Program, Naval Health Research Center, San Diego, California, United States of America; 5 Infectious Disease Clinic, Naval Medical Center, San Diego, California, United States of America; 6 Infectious Disease Service, Walter Reed Army Medical Center, Washington, District of Columbia, United States of America; 7 Division of Infectious Diseases, National Naval Medical Center, Bethesda, Maryland, United States of America; 8 Division of Infectious Diseases, Naval Medical Center, Portsmouth, Virginia, United States of America; University of Cape Town, South Africa

## Abstract

**Background:**

Factors associated with serologic hepatitis B virus (HBV) outcomes in HIV-infected individuals remain incompletely understood, yet such knowledge may lead to improvements in the prevention and treatment of chronic HBV infection.

**Methods and Findings:**

HBV-HIV co-infected cohort participants were retrospectively analyzed. HBV serologic outcomes were classified as chronic, resolved, and isolated-HBcAb. Chronic HBV (CHBV) was defined as the presence of HBsAg on two or more occasions at least six months apart. Risk factors for HBV serologic outcome were assessed using logistic regression. Of 2037 participants with HBV infection, 281 (14%) had CHBV. Overall the proportions of HBV infections classified as CHBV were 11%, 16%, and 19% for CD4 cell count strata of ≥500, 200–499, and <200, respectively (*p*<0.0001). Risk of CHBV was increased for those with HBV infection occurring after HIV diagnosis (OR 2.62; 95% CI 1.78–3.85). This included the subset with CD4 count ≥500 cells/µL where 21% of those with HBV after HIV diagnosis had CHBV compared with 9% for all other cases of HBV infection in this stratum (*p* = 0.0004). Prior receipt of HAART was associated with improved HBV serologic outcome overall (*p* = 0.012), and specifically among those with HBV after HIV (*p* = 0.002). In those with HBV after HIV, HAART was associated with reduced risk of CHBV overall (OR 0.18; 95% CI 0.04–0.79); including reduced risk in the subsets with CD4 ≥350 cells/µL (*p*<0.001) and CD4 ≥500 cells/µL (*p* = 0.01) where no cases of CHBV were seen in those with a recent history of HAART use.

**Conclusions:**

Clinical indicators of immunologic status in HIV-infected individuals, such as CD4 cell count, are associated with HBV serologic outcome. These data suggest that immunologic preservation through the increased use of HAART to improve functional anti-HBV immunity, whether by improved access to care or earlier initiation of therapy, would likely improve HBV infection outcomes in HIV-infected individuals.

## Introduction

Liver-related complications have become an increasingly important cause of morbidity and mortality in HIV-infected patients since the advent of highly active antiretroviral therapy (HAART).[1], [2], [3], [4], [5], [6] Despite the high prevalence of HBV co-infection among individuals with HIV and its impact on management and clinical outcomes, the optimal strategies for the prevention and treatment of chronic HBV infection (CHBV) in those with HIV are not well defined.

HBV vaccination of susceptible individuals is recommended as a means of primary prevention,[7] but the clinical effectiveness of HBV vaccine in this population when given after HIV diagnosis may be limited,[8] and as many as 20% of HBV infections in HIV-infected individuals may be chronic.[9], [10] In a small study of HBV/HIV co-infected individuals from the pre-HAART era, the mean CD4 cell count was lower for the seven subjects with CHBV compared with the 24 subjects that cleared HBsAg.[9] Thus, in the absence of an effective vaccine for primary prevention, preservation of higher CD4 cell counts and anti-HBV immunity during the course of HIV may be associated with reduced risk of becoming chronically infected with HBV. Therefore, either improved access to or earlier initiation of HAART may provide an effective method for prevention of HBV associated disease in this patient population.

In addition to informing possible prevention practices for HBV, an evaluation of clinical associations with HBV serologic outcomes may help indirectly inform treatment strategies for HBV in patients with HBV/HIV co-infection. Current HIV-treatment guidelines recommend initiation of HAART for HBV/HIV co-infected adults when HBV treatment is indicated [11], [12] although supporting data are very limited. Some have hypothesized that HAART may improve rates of response to anti-HBV therapy.[13] Supporting this hypothesis, improvement of anti-HBV immunity with ritonavir monotherapy[14], and seroconversion from HBsAg to HBsAb following initiation of HAART have been reported.[15] In addition, previous investigations have found associations between CD4 responses to HAART with HBV viral suppression[16] and possibly HBV seroconversion.[17] However, the conclusions from these studies regarding the impact of HAART upon improvement of HBV serologic markers were limited because few individuals cleared HBsAg and most were receiving HAART. Because of these limitations, better understanding of the determinants of HBV serologic outcome may suggest possible improvements in the care of those with chronic HBV/HIV co-infection, such as the addition of HAART to anti-HBV therapy.

Therefore, to inform future HBV prevention and treatment strategies we sought to evaluate the associations between the risk of CHBV and the timing of HBV infection relative to HIV diagnosis (before or after), CD4 cell count at the time of HBV diagnosis, and HAART use preceding HBV diagnosis. We hypothesized that such factors, reflective of anti-HBV immunity at the time of HBV infection or diagnosis, would be associated with the subsequent risk of developing chronic compared to resolved HBV infection in co-infected individuals.

## Methods

### Study Cohort

The U.S. Military HIV Natural History Study (NHS) is an ongoing, continuous enrollment observational cohort of HIV-infected Department of Defense (DoD) beneficiaries followed at seven participating military medical centers in the United States and has been previously described.[8] Enrolling since 1986, the NHS has approximately 5000 participants with signed, written consent. Patients are seen every six months following enrollment. Data collected at each visit include demographic information, past and interim medical history, medications, vaccinations, and standard clinical laboratory studies. HIV exposure category is not routinely captured, however rates of HIV risk behaviors have been previously reported and intravenous drug use is rare (<1%).[18]

### Ethics Statement

All adult DoD beneficiaries with a diagnosis of HIV infection followed at a participating site and able to provide consent were eligible for participation in the NHS. Approval for this research was obtained from the institutional review board at each participating site.

### Participant Selection and Definitions

All NHS participants with a documented date of HIV seropositivity through 2008, and serologic diagnosis of HBV infection as defined below were included in the current analyses. HBV infection and serologic outcomes were defined using the following non-overlapping categories:

1. Chronic HBV infection (CHBV), defined as ever having reactive HBsAg on two or more separate occasions at least six months apart,
2. Isolated HBcAb (IcHBV), defined as having reactive HBcAb on two or more separate occasions, and tested for both HBsAg and HBsAb and never positive for HBsAg nor HBsAb, or
3. Resolved HBV infection (RHBV), defined as having reactive HBcAb and HBsAb, and never meeting criteria for CHBV.

All cases of HBV infection were also sub-classified into one of two groups *a priori* based upon the timing of HBV diagnosis relative to HIV diagnosis. Participants initially non-reactive for both HBsAg and HBcAb on or after the date of HIV infection but later meeting criteria for HBV infection were classified as having HBV infection definitively after HIV diagnosis; those with HBV diagnosis at the time of HIV diagnosis and those testing positive for HBV after HIV but missing previous negative HBsAg or HBcAb results (i.e. unknown timing of HBV infection relative to HIV diagnosis) were grouped together as HBV infections which did not definitively occur after HIV.

Mono/dual antiretroviral therapy (ART) and highly active antiretroviral therapy (HAART) use was categorized based on use within the 12 months preceding HBV diagnosis. HAART was defined as a combination of at least three antiretroviral agents, similar to previous investigations.[19] HBV-active mono/dual ART or HAART was defined as ART or HAART containing lamivudine, emtricitibine, or tenofovir. The presence of an AIDS-defining illness was defined using 1993 CDC criteria,[20] with the exception of isolated CD4 count <200 cells/µL. Infection with syphilis, *N. gonorrhea*, *C. trachomatis*, or genital herpes simplex virus was considered to be a sexually transmitted infection (STI).

### Study Design and Statistical Methods

Descriptive statistics were used to examine the characteristics of participants with HBV infection overall and by HBV outcome category. Medians were reported with inter-quartile ranges (IQR). Proportions were compared with chi-square or Fisher's Exact tests as appropriate. Continuous variables were compared using the Kruskal-Wallis test. Univariate and multivariate logistic regression models were used to assess factors associated with IcHBV compared with RHBV, and CHBV compared with RHBV. Subset analyses evaluating factors associated with CHBV in those with HBV infection definitively after HIV diagnosis were also performed. For analyses regarding use of HAART in individuals with HBV definitively after HIV, participants were sub-grouped by non-exclusive CD4 categories of ≥350 and ≥500 cells/µL. Initial multivariate models included variables captured for at least 75% of participants with *p*≤0.10 in univariate analysis. Final multivariate models incorporated adjustments for factors which were different between HBV diagnoses before 1996 and HBV diagnoses in 1996 or later. To further explore the interactions between CD4 cell count, timing of HBV diagnosis relative to HIV diagnosis, and HAART use, we classified subjects into mutually exclusive clinically relevant categories based on their CD4 count at the time of HBV diagnosis (<200, 200–499, or ≥500 cells/µL). Differences in the proportion of participants with CHBV and RHBV across CD4 categories were examined using the Cochran-Armitage test for trend. Odds ratios (OR) were reported with 95% confidence intervals (CI). All *p*-values were two-sided, and all statistical analyses were performed using SAS software (version 9.1, Cary, NC).

## Results

A total of 2,037 participants met criteria for HBV infection, and were included overall. (Table 1) The median number of HBV serologic panel assessments was 7 (IQR 4-11) overall; 8 (IQR 5-12) for those with CHBV, 5 (IQR, 3–8) for those with IcHBV, and 7 (3–11) for those with RHBV. Of those classified as having CHBV, 255 (91%) were persistently reactive for HBsAg beginning at the time of HBV diagnosis and 26 (9%) were reactive for HBV serologic markers other than HBsAg at the time of HBV diagnosis and later became persistently reactive for HBsAg. Of those classified as having RHBV, 1233 (79%) were reactive concurrently for HBcAb and HBsAb at the time of HBV diagnosis and never became chronically infected, and 330 (21%) had initial serologic evidence of HBV infection without HBsAb, never became chronically infected, and later developed HBsAb. A total of 407 (20%) HBV infections were diagnosed after 1995. Differences in characteristics at the time of HBV diagnosis between those with HBV diagnosed during the pre-HAART era compared with the HAART era included age (*p*<0.01), gender (*p* = 0.03), known HIV seroconversion status (*p*<0.01), and ethnicity (*p* = 0.06), whereas median CD4 cell count at HBV diagnosis was similar for the pre-HAART and HAART eras (474 vs. 443, respectively; *p* = 0.20). Seventy-nine percent of participants had not received any HIV therapy in the year prior to HBV diagnosis. Two percent of the participants who received only ART (7/339) and 91% of those who received HAART (79/87) received HBV-active agents as a component of therapy. Interferon therapy was rare in all HBV serologic outcome categories. Five (1.8%) of those who developed CHBV, 3 (1.6%) of those who developed IcHBV, and 30 (1.9%) of those who developed RHBV received interferon treatment following HBV diagnosis.

**Table 1:** Characteristics of participants at the time of HBV diagnosis by HBV infection outcome.

Characteristic	N	CHBV (N = 281)	IcHBV (N = 193)	RHBV (N = 1563)
**HIV Seroconverter, N (%)**	2037	132 (47)	67 (35)	838 (54)
**Age, years, median (IQR)**	2037	31 (27–35)	34 (28–37)	32 (27–37)
**Years from HIV to HBV diagnosis, median (IQR)**	2037	1.2 (0.1–3.5)	1.4 (0.1–4.0)	0.9 (0.1–3.2)
**Male, N (%)**	2037	275 (98)	183 (95)	1524 (98)
**Self-identified ethnicity, N (%)**				
Caucasian	885	112 (40)	72 (37)	701 (45)
African American	950	147 (52)	104 (54)	699 (45)
Hispanic/Puerto Rican/Mexican	153	18 (6)	11 (6)	124 (8)
Other	49	4 (1)	6 (3)	39 (3)
**CD4 cell count, cells/µL, median (IQR)**	1979	413 (272–578)	434 (293–629)	485 (320–664)
**HIV RNA, log_10_copies/mL, median (IQR)**	571	4.3 (3.9–4.7)	4.2 (3.6–4.9)	4.2 (3.4–4.7)
**AIDS-Defining Illness, N (%)**	2037	16 (6)	13 (7)	81 (5)
**Anti-HCV prior to HBV, N (%)**	230	4 (9)	2 (15)	11 (7)
**STI prior to HBV, N (%)**	2021	141 (51)	104 (55)	796 (51)
**ART use prior to HBV** a **, N (%)**				
None	1604	211 (75)	149 (77)	1244 (80)
HBV inactive mono/dual ART	339	62 (22)	40 (21)	237 (15)
HBV active mono/dual ART	7	2 (1)	1 (1)	4 (1)
HBV inactive HAART	8	1 (1)	0 (0)	7 (1)
HBV active HAART	79	5 (2)	3 (2)	71 (5)
**HBV vaccination doses, N (%)**				
None	1783	247 (88)	171 (89)	1365 (87)
1–2	145	23 (8)	12 (6)	110 (7)
≥3	109	11 (4)	10 (5)	88 (6)
**HBV vaccination relative to HIV, N (%)**				
None	1783	247 (88)	171 (89)	1365 (87)
All doses prior to HIV	82	6 (2)	4 (2)	72 (5)
Doses before and after HIV	32	2 (1)	3 (2)	27 (2)
All doses after HIV	140	26 (9)	15 (8)	99 (6)
**HBV after HIV, N (%)**	2037	60 (21)	22 (11)	169 (11)

a Within the year prior to HBV diagnosis.

CHBV, chronic hepatitis B virus infection; IcHBV, Isolated HBcAb hepatitis B virus infection; RHBV, resolved hepatitis B virus infection; HBV, hepatitis B virus; HCV, hepatitis C virus; HIV, human immunodeficiency virus; ART, antiretroviral therapy; HAART, highly active antiretroviral therapy; STI, sexually transmitted infection.

### Factors Associated with IcHBV and CHBV Overall

Results of univariate and multivariate analyses of factors associated with IcHBV or CHBV compared with RHBV are provided separately (Text S1).

### HBV Infection Serologic Outcome by CD4 Cell Count Categories

**Figure 1:**
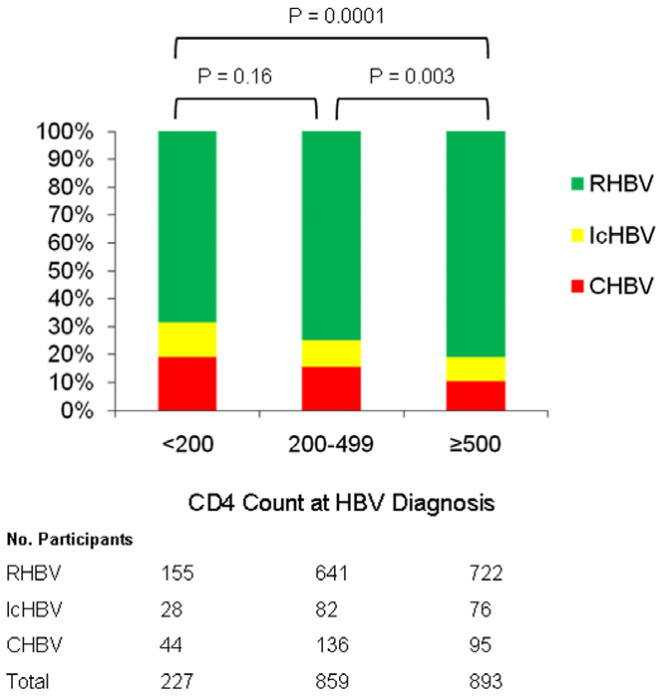
HBV infection outcome by CD4 cell count strata. (CHBV, chronic hepatitis B virus infection; IcHBV, Isolated HBcAb hepatitis B virus infection; RHBV, resolved hepatitis B virus infection).

Those with HBV diagnosis when the CD4 count was ≥500 had significantly different serologic outcomes than those with HBV diagnosis when CD4 counts were either 200–499 (*p* = 0.003) or <200 (*p* = 0.0001) (Fig. 1). Furthermore, the proportion of participants with CHBV increased as the CD4 cell count at HBV diagnosis decreased (11%, 16%, and 19% for CD4 cell count strata of ≥500, 200–499, and <200, respectively; *p*<0.0001), whereas the proportion of participants with RHBV increased as CD4 cell count at the time of HBV infection increased (68%, 75%, and 81% for CD4 cell count strata of <200, 200–499, and ≥500, respectively; *p*<0.0001).

### HBV Infection Serologic Outcome by Onset of HBV Infection Relative to HIV Diagnosis

**Figure 2:**
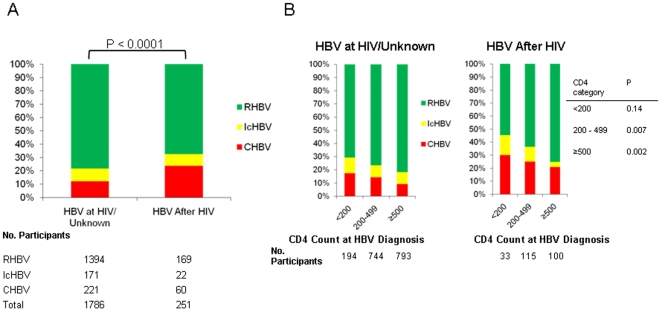
HBV infection outcome by timing of HBV infection relative to HIV diagnosis. (Panel A, Overall; Panel B, by CD4 cell count strata) (CHBV, chronic hepatitis B virus infection; IcHBV, Isolated HBcAb hepatitis B virus infection; RHBV, resolved hepatitis B virus infection).

For those with HBV after HIV, 24% of infections were CHBV compared with 12% for all other HBV infections (i.e. those diagnosed concurrently with HBV and HIV, and those with unknown timing of HBV infection relative to HIV) (*p*<0.0001) (Fig. 2A). Furthermore, those with HBV infection following HIV diagnosis had different HBV serologic outcomes by CD4 category compared with those who were HBV infected but not definitively infected with HBV after HIV (Fig. 2B). Even in the subgroup with CD4 count ≥500 at the time of HBV diagnosis, those with HBV after HIV were more likely to have CHBV (21% compared with 9%, *p* = 0.0004).

### HBV Infection Serologic Outcome and Receipt of Antiretroviral Therapy

**Figure 3:**
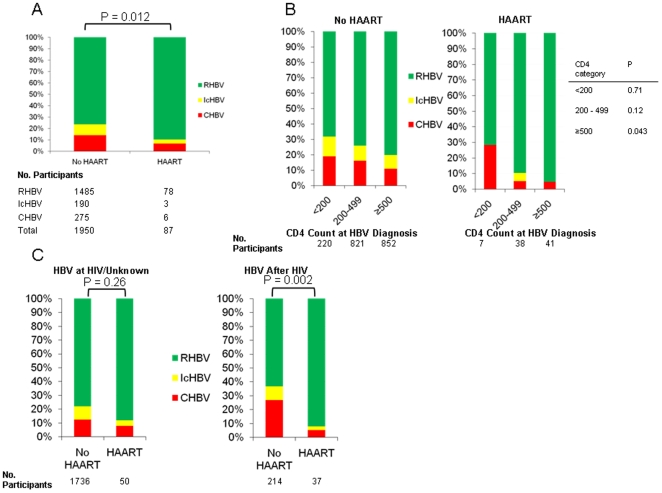
HBV infection outcome by ART use prior to HBV diagnosis. (Panel A, Overall; Panel B, by CD4 cell count strata; Panel C, by timing of HBV infection relative to HIV diagnosis). (CHBV, chronic hepatitis B virus infection; IcHBV, Isolated HBcAb hepatitis B virus infection; RHBV, resolved hepatitis B virus infection).

Individuals who received HAART in the year prior to HBV diagnosis had significantly different serologic outcomes than those who did not receive HAART prior to HBV diagnosis (*p* = 0.012; Fig. 3A). Regarding HBV active therapy, the proportion of participants with CHBV was not significantly lower for those receiving HBV-active ART (29%) compared with those on HBV-inactive ART (18%; *p* = 0.62) although only seven participants were in the former category. Additionally, proportions of CHBV were similar for those on HAART either with or without HBV-active agents (6% vs. 13%, respectively; *p* = 0.45), although only eight participants received HBV-inactive HAART.

In evaluating HBV serologic outcomes by HAART receipt using CD4 cell count strata of <200, 200–499, and ≥500 cells/µL, outcomes were improved in those receiving HAART but outcomes were only significantly improved in the ≥500 cells/µL stratum. (Fig. 3B) In addition, for the subset of those with CD4 cell count ≥350 cells/µL at HBV diagnosis, 2/61 (3%) who received HAART developed CHBV compared with 171/1309 (13%) who did not receive HAART (*p* = 0.02).

Finally, the association between improved HBV outcomes and receipt of HAART was strongest for participants with HBV infection after HIV diagnosis (Fig. 3C). For individuals with HBV infection after HIV diagnosis, 2/37 (5%) receiving HAART prior to HBV diagnosis developed CHBV compared with 58/214 (27%) for those not receiving HAART (*p* = 0.003). Among those with HBV after HIV, receipt of HAART was associated with an 82% reduction in risk of developing CHBV (adjusted OR 0.18; 95% CI 0.04–0.79). Lastly, for those with HBV after HIV and with CD4 count ≥350, none of those on HAART developed CHBV compared with 39 (28%) of those not on HAART (*p*<0.001); similarly, among those with CD4 count ≥500, none of those on HAART developed CHBV compared with 21 (26%) of those not on HAART (*p* = 0.01).

## Discussion

Our investigation regarding factors associated with HBV serologic outcome in HIV-infected adults both confirms and expands upon previous findings. While the majority of all HBV infections in our study were resolved, over one quarter of HBV/HIV co-infected participants had some form of unresolved infection. Furthermore, approximately 20% of HBV infections occurring after HIV diagnosis were chronic regardless of CD4 cell count at the time of HBV diagnosis. These observations emphasize the importance of HBV prevention for all patients with HIV, including those with high CD4 cell counts. Our findings also highlight the association between functional immune status at the time of HBV infection and subsequent HBV serologic outcome, and suggest that improved functional anti-HBV immunity through maintenance of higher CD4 cell counts and increased use of HAART would likely improve serologic HBV outcomes in HIV-infected individuals.

From a simplified perspective the immune response to HBV infection occurs in two phases: phase one, the response to acute HBV exposure, and phase two, the response required to maintain immunologic control of HBV within hepatocytes indefinitely. In both phases strong, polyclonal, multi-specific CD8 T cells appear to be the primary component, although CD4 T cells serve a critical role in supporting such responses.[21], [22], [23] Therefore, interventions to improve HBV serologic outcomes may target one or both phases of this response. The importance of cellular immune function in determining the serologic outcome from HBV infection is supported by several observations from our study including an increased risk of CHBV in those with HBV infection after HIV diagnosis and those with lower CD4 cell counts at the time of HBV diagnosis, as well as an increased likelihood of RHBV in those with higher CD4 cell counts, and improvements in HBV outcome for those infected with HBV while receiving HAART. The central importance of cellular immune function in determining HBV serologic status is also supported by a smaller previous study which similarly found those with CHBV had lower mean CD4 cell counts, and the proportion of CHBV infections in those with HBV after HIV was high, approximately 20%.[9]

Our findings also demonstrate the complex relationship between HBV serologic outcomes and the degree of immunologic function during either phase of the immune response to HBV. While CD4 cell count at the time of HBV diagnosis was inversely related to the risk of CHBV, such risk was also altered by whether or not HBV infection occurred after HIV diagnosis. In other words, an individual infected with HBV prior to HIV whose CD4 count later declined had lower risk of CHBV than an individual infected with HBV after HIV diagnosis with a similar CD4 cell count. Therefore, our study suggests a significant portion of HBV serologic outcome is determined by an individual's ability to mount an effective immune response at the time of HBV exposure, regardless of future alterations in immune status. HBV serologies have been reported to fluctuate over time in HIV-infected individuals which could potentially limit conclusions from our study in this regard.[24], [25] However, even though we did not specifically evaluate longitudinal changes in HBV serologies, 91% of individuals we classified as having CHBV were persistently reactive for HBsAg from the date of HBV diagnosis further suggesting that CHBV is established early in the course of HBV infection. This observation also supports our design evaluating the associations between factors known at the time of HBV diagnosis with subsequent HBV serologic outcome. Additionally, true HBV reverse seroconversion in HIV-infected individuals is reportedly very rare.[26] Therefore, we conclude that immunologic events during phase one of the response to HBV infection are the crucial determinants of HBV serologic outcome. Considering our results together with previous studies reporting low rates of HBsAg clearance over time in those with HBV/HIV co-infection,[16], [17], [27], [28], [29] those infected with CHBV after HIV infection may have reduced ability to ever develop adequate immunity to control HBV, or develop resolved HBV infection.

The high proportion of CHBV in those with HIV, including those with CD4 cell count ≥500, combined with the observation that long-term HBV serologic outcome may be determined at the time of HBV exposure, further emphasizes the importance of HBV prevention in this patient population. Notably, those with HBV infection after HIV diagnosis who received HAART immediately preceding HBV diagnosis were less likely to have CHBV than those not receiving HAART. For such individuals on HAART the proportion of those with CHBV was 5%, similar to HIV-negative individuals infected with HBV as adults.[10] This observation suggests HAART is capable of reversing the HIV-associated dysfunction of anti-HBV CD8 and CD4 T cells required for a successful response to acute HBV exposure. Furthermore, the ability of HAART use to alter HBV outcome remained evident in the subset of participants with CD4 cell count ≥500. Not surprisingly, HAART appeared to have little or no benefit for those who were likely infected with HBV before HIV diagnosis.

As the second phase of the immune response to HBV infection involves similar CD8 and CD4 T cell responses to those seen after acute HBV exposure[21] and because a significant portion of our participants developed RHBV (a potential goal of CHBV treatment), our findings may also have relevance regarding the treatment of CHBV in individuals with HIV. As a cross-sectional study evaluating HBV serologies and not clinical outcomes, our analysis was not designed to evaluate the optimal treatment of CHBV. However, two previous cohort investigations suggested that HAART may improve HBV-related treatment outcomes including HBV viral suppression and seroconversion.[16], [17] In addition, small studies of HBV/HIV co-infected individuals have demonstrated reconstitution of anti-HBV CD8 and CD4 T cell responses following HAART initiation in those with RHBV,[30], [31] further supporting the concept that HAART may improve the second phase of the immune response to HBV, thereby increasing HBV treatment success. Our study provides indirect support of current HIV treatment guideline recommendations,[11], [12] suggesting the use of HAART may improve serologic responses to HBV treatment indirectly through improvements in anti-HBV immunity, including the subset of patients with high CD4 cell counts. Previous studies have also shown low rates of HBeAg or HBsAg seroconversion with HBV mono- or dual therapy in HIV-infected individuals.[32,33,34] Therefore, the optimal treatment approach for HBV infection in HIV-infected individuals may indeed be anti-HBV therapy as a component of HAART.

The optimal strategies for prevention and treatment of HBV in HIV-infected individuals remain to be defined. From our investigation, HBV serologic outcomes were significantly related to several factors reflecting the functional immune status of an individual. Future investigations will need to evaluate such associations with clinical outcomes. Evidence and support for the earlier initiation of and improved access to HAART continue to increase,[32], [33] and an additional benefit associated with HAART administration in those with both low and high CD4 cell counts may be reduced risk of CHBV. This reduced risk may result from improved function of the factors necessary for a successful immune response to HBV, in addition to direct anti-HBV effects. Therefore, increased use of the currently recommended first-line HAART regimens may significantly reduce the development of CHBV following HBV exposure in HIV-infected individuals.

## Supporting Information

Text S1 (0.11 MB DOC)
