# Supplementary material for: The Effect of Human Immunodeficiency Virus on Hepatitis B Virus Serologic Status in Co-Infected Adults
Source: PLoS One. 2010 Jan 13;5(1):e8687. doi: 10.1371/journal.pone.0008687 (PMC2800198; doi:10.1371/journal.pone.0008687)
Supplement: Text S1 — (0.11 MB DOC) [file pone.0008687.s001.doc]

09-PONE-RA-11878R1

Supporting Information

RESULTS

**Factors Associated with IcHBV and CHBV Overall.** Univariate and final multivariate analysis of factors associated with IcHBV compared with RHBV are shown in Table S1. From the final multivariate model adjusted for age, gender, and HIV seroconversion status to account for differences in HBV diagnosis era, African American compared with Caucasian ethnicity was associated with significantly increased risk of IcHBV (OR 1.55; 95% CI 1.12-2.14). Receipt of HAART was also associated with reduced risk although the association did not reach statistical significance (OR 0.31; 95% CI 0.09-1.06).

From the final adjusted multivariate model evaluating factors associated with CHBV overall, the risk of CHBV decreased by 11% for every 100 cells/μL increase in CD4 cell count. (Table S2) In addition, HBV infection after HIV diagnosis was associated with increased risk of CHBV (OR 2.62, 95% CI 1.78-3.85), as was African American compared with Caucasian ethnicity (OR 1.35, 95% CI 1.02-1.79). Use of HAART was associated with reduced risk of CHBV (OR 0.41; 95% CI 0.17-1.01) although this did not reach statistical significance. Subcategories for HBV-active ART and HAART were not used in the multivariate model because few received either HBV-active ART or HBV-inactive HAART.

Table S1. Univariate and multivariate analysis of factors at the time of HBV diagnosis associated with Isolated HBcAb (IcHBV) compared with Resolved HBV infection (RHBV).

| **Characteristic** | **Univariate**  **OR (95% CI)** | **P** | **Multivariate^a^**  **OR (95% CI)** | **P** |
| --- | --- | --- | --- | --- |
| **Self-identified ethnicity** |  |  |  |  |
| Caucasian | Referent |  | Referent |  |
| African American | 1.45 (1.05-1.99) | 0.02 | 1.55 (1.12-2.14) | 0.01 |
| Hispanic/Puerto Rican/Mexican | 0.86 (0.45-1.68) | 0.67 | 0.86 (0.44-1.67) | 0.65 |
| Other | 1.50 (0.61-3.66) | 0.38 | 1.53 (0.62-3.81) | 0.36 |
| **CD4 cell count, per 100 cells/μL increase** | 0.96 (0.91-1.01) | 0.13 |  |  |
| **HIV RNA , per 1.0 log_10_copies/mL increase** | 1.09 (0.75-1.57) | 0.66 |  |  |
| **Prior AIDS-Defining Illness** | 1.32 (0.72-2.42) | 0.37 |  |  |
| **Anti-HCV prior to HBV** | 2.63 (0.52-13.36) | 0.24 |  |  |
| **STI prior to HBV** | 1.13 (0.84-1.53) | 0.41 |  |  |
| **ART use prior to HBV^b^** |  |  |  |  |
| None | Referent |  | Referent |  |
| Mono/dual therapy | 1.42 (0.98-2.06) | 0.06 | 1.18 (0.80-1.75) | 0.40 |
| HAART | 0.32 (0.10-1.03) | 0.06 | 0.31 (0.09-1.06) | 0.06 |
| **HBV vaccination doses** |  |  |  |  |
| None | Referent |  |  |  |
| 1-2 doses | 0.87 (0.47-1.61) | 0.66 |  |  |
| ≥3 doses | 0.91 (0.46-1.78) | 0.78 |  |  |
| **HBV vaccination relative to HIV** |  |  |  |  |
| None | Referent |  | Referent |  |
| All doses prior to HIV | 0.44 (0.16-1.21) | 0.11 | 0.66 (0.23-1.86) | 0.43 |
| Doses before and after HIV | 0.90 (0.27-2.99) | 0.86 | 1.55 (0.45-5.34) | 0.49 |
| All doses after HIV | 1.25 (0.71-2.19) | 0.44 | 1.67 (0.91-3.04) | 0.10 |
| **HBV after HIV, N(%)** | 1.06 (0.66-1.70) | 0.81 |  |  |

^a^Adjusted for age at HBV diagnosis, gender, and known HIV seroconversion status.

^b^Within the year prior to HBV diagnosis.

IcHBV, Isolated HBcAb hepatitis B virus infection; RHBV, resolved hepatitis B virus infection HBV, hepatitis B virus; HCV, hepatitis C virus; HIV, human immunodeficiency virus; ART, antiretroviral therapy; HAART, highly active antiretroviral therapy; STI, sexually transmitted infection.

Table S2. Univariate and multivariate analysis of factors at the time of HBV diagnosis associated with Chronic HBV infection (CHBV) compared with Resolved HBV infection (RHBV).

| **Characteristic** | **Univariate**  **OR (95% CI)** | **P** | **Multivariate^a^**  **OR (95% CI)** | **P** |
| --- | --- | --- | --- | --- |
| **Self-identified ethnicity** |  |  |  |  |
| Caucasian | Referent |  | Referent |  |
| African American | 1.32 (1.01-1.72) | 0.04 | 1.35 (1.02-1.79) | 0.03 |
| Hispanic/Puerto Rican/Mexican | 0.91 (0.53-1.55) | 0.72 | 0.98 (0.57-1.69) | 0.94 |
| Other | 0.64 (0.23-1.83) | 0.41 | 0.72 (0.25-2.10) | 0.55 |
| **CD4 cell count, per 100 cells/μL increase** | 0.89 (0.85-0.94) | <0.0001 | 0.89 (0.84-0.94) | <0.0001 |
| **HIV RNA , per 1.0 log_10_copies/mL increase** | 1.26 (0.93-1.70) | 0.14 |  |  |
| **Prior AIDS-Defining Illness** | 1.10 (0.64-1.92) | 0.72 |  |  |
| **Anti-HCV prior to HBV** | 1.34 (0.41-4.43) | 0.63 |  |  |
| **STI prior to HBV** | 0.97 (0.75-1.25) | 0.81 |  |  |
| **ART use prior to HBV^b^** |  |  |  |  |
| None | Referent |  | Referent |  |
| Mono/dual therapy | 1.57 (1.15-2.14) | <0.01 | 1.07 (0.75-1.52) | 0.71 |
| HAART | 0.45 (0.20-1.05) | 0.07 | 0.41 (0.17-1.01) | 0.05 |
| **HBV vaccination doses** |  |  |  |  |
| None | Referent |  |  |  |
| 1-2 doses | 1.16 (0.72-1.85) | 0.55 |  |  |
| ≥3 doses | 0.69 (0.36-1.31) | 0.26 |  |  |
| **HBV vaccination relative to HIV** |  |  |  |  |
| None | Referent |  | Referent |  |
| All doses prior to HIV | 0.45 (0.19-1.05) | 0.06 | 0.55 (0.23- 1.30) | 0.17 |
| Doses before and after HIV | 0.41 (0.10-1.72) | 0.22 | 0.40 (0.09-1.76) | 0.22 |
| All doses after HIV | 1.51 (0.96-2.37) | 0.07 | 0.99 (0.58-1.70) | 0.96 |
| **HBV after HIV, N(%)** | 2.24 (1.61-3.11) | <0.0001 | 2.62 (1.78-3.85) | <0.0001 |

^a^Adjusted for age at HBV diagnosis, gender, and known HIV seroconversion status.

^b^Within the year prior to HBV diagnosis.

CHBV, chronic hepatitis B virus infection; RHBV, resolved hepatitis B virus infection HBV, hepatitis B virus; HCV, hepatitis C virus; HIV, human immunodeficiency virus; ART, antiretroviral therapy; HAART, highly active antiretroviral therapy; STI, sexually transmitted infection.
